# Epidemiological and clinical characteristics of children and young adults with Glanzmann’s thrombasthenia in upper Egypt: a multicenter cross-sectional study

**DOI:** 10.1007/s00277-025-06290-5

**Published:** 2025-03-13

**Authors:** Gehan Lotfy Abdel Hakeem Khalifa, Amr Abdallah El-Sayed, Zahraa Elmasry, Khalid I. Elsayh, Zizi T. Atwa, Dalia Saber Morgan, Ebtesam Esmail Hassan, Mohmed A. Hassan, Mervat A.M. Youssef

**Affiliations:** 1 Pediatric Hematology Unit, Department of Pediatrics, Faculty of Medicine, Minia University, Al-Minya, Egypt; 2 Medical Affairs Department, Novo Nordisk Egypt, Cairo, Egypt; 3 Freelance Public Health Researcher, Cairo, Egypt; 4 Pediatric hematology unit, Department of Pediatrics, Faculty of Medicine, Sohag University, Sohag, Egypt; 5 Pediatric hematology unit, Department of Pediatrics, Faculty of Medicine, Assiut University, Assiut, Egypt; 6 Pediatric hematology unit, Department of Pediatrics, Faculty of Medicine, Fayoum University, Al-Fayoum, Egypt; 7 Pediatric hematology unit, Department of Pediatrics, Faculty of Medicine, Beni-Suef University, October 6 University, Beni-Suef, Cairo, Egypt; 8 Department of Public Health and Preventive Medicine, Faculty of Medicine, Minia University, Al-Minya, Egypt; 9 Genetics Unit, Department of Pediatrics, Faculty of Medicine, Assiut University, Assiut, Egypt

**Keywords:** Glanzmann’s thrombasthenia, Inherited platelet disorders, Rare bleeding disorders, Egypt, Epidemiological study, Cross-sectional study

## Abstract

**Background:**

Glanzmann’s thrombasthenia (GT) is an inherited rare bleeding disorder characterized by a deficiency or functional defect in the platelet αIIbβ3 integrin. This impairs normal platelet aggregation and leads to prolonged and spontaneous mucocutaneous bleeds.

**Objectives:**

To report disease characteristics of a GT cohort from five tertiary hospitals in Upper Egypt.

**Materials and methods:**

We conducted a retrospective cross-sectional observational study, relying on patients’ medical records and interview surveys to collect information from patients diagnosed with congenital GT between October 2023 and April 2024.

**Results:**

We recruited 131 people with GT (PwGT) of different ages, mainly children and adolescents. 73.3% of the study cohort had type I GT, 23.7% had type II GT, and 3% had type III GT. Consanguinity and family history were prevalent in our cohort, with an expected prevalence of more than one per 200,000 in our region. The median value of ADP aggregation was 8%. In type I GT, the median levels of CD41 and CD61 were 0.3%. In contrast, type II GT had median levels of 12% for CD41 and 17% for CD61. The most frequent manifestations were epistaxis (77.1%), subcutaneous bleeds (40.5%), menorrhagia (22.1%), and mucosal bleeds (18.3%). 72.5% of PwGT used rFVIIa and 69.5% used platelet transfusions to treat acute and surgical bleeds, while only 6.9% used tranexamic acid as monotherapy.

**Conclusion:**

Estimating the actual burden of GT in Egypt requires accurate diagnoses, as well as systematic and standardized data collection. The rooted consanguinity pattern in Upper Egypt contributes to a higher prevalence of GT above the country’s average.

## Introduction

Glanzmann’s thrombasthenia (GT) is a rare genetic autosomal recessive bleeding disorder characterized by a deficiency or functional defect in the platelet surface glycoprotein (GP) IIb-IIIa receptor complex, also known as the CD41-CD61 receptor complex located on the platelet surface [1–4] due to genetic mutations in the ITGA2B and ITGB3 genes that are situated adjacently on region two of the long arm of chromosomes 17 [5–7]. This receptor complex is a subtype of the GP receptors, called integrin with αIIb and β3 subunits, which binds platelets together via fibrinogen to form platelet aggregates [7–10]. Quantitative or qualitative defects in the integrin αIIbβ3 result in impaired platelet aggregation, insufficient clot formation, and prolonged bleeding manifestations [1–4]. Clinical manifestations of GT entail moderate to severe prolonged and spontaneous bleeding from the mucocutaneous tissues, such as epistaxis, gingival bleeding, menstrual bleeding, and, to a lesser extent, gastrointestinal bleeding, with lifelong bleeding starting in childhood [1–4]. Easy bruising, petechiae, purpura, and ecchymosis may occur due to minor trauma, and excessive bleeding is experienced after trauma or surgical and dental procedures [11–14]. Intracranial hemorrhage, hematuria, hemarthrosis, and organ bleeding are rare [1–3]. GT is classified into three types based on the expression and function of the integrin αIIbβ3: Type I GT, in which αIIbβ3 levels are < 5% of normal; Type II GT, in which αIIbβ3 levels range from 5% to ≤ 25% of normal; and Type III, or variant-type GT, in which αIIbβ3 levels are > 50% of normal but non-functioning [1–4, 13–18]. Epidemiological and clinical characteristics of people with Glanzman thrombasthenia (PwGT) have been previously reported from several parts of the world [11, 13, 14, 19–34]. On the other hand, published reports from Egypt are scarce [35]. In addition, the Upper Egypt region, extending from Giza governorate in the north to Aswan governorate in the south along the Nile River was inhabited by 38.9% of the country’s population in 2023 [36]. Therefore, it would be of value to map the disease characteristics in the local GT population in Upper Egypt. Accordingly, this study aimed to report the epidemiological, sociodemographic, diagnostic, and clinical characteristics of a GT cohort from five University hospitals in five governorates in Upper Egypt.

## Materials and methods

### Study design and data collection

We conducted a retrospective cross-sectional observational study, collecting information from people diagnosed with congenital GT attending pediatric hematology units at five tertiary hospitals (Fayoum, Beni-Suef, Minia, Assiut, and Sohag) in Upper Egypt between October 2023 and April 2024. Study information was collected from the patient’s medical records and through interview surveys with PwGT and their caregivers during treatment visits or via phone calls. These surveys were completed by the treating physician during face-to-face interviews with PwGT and their caregivers while attending the hospital for episodic treatment. Before participation, informed verbal consent and/or assent was obtained from PwGT and their caregivers.

### Diagnosis and classification of GT types

The diagnosis of GT in our cohort was initially suspected based on clinical manifestations and confirmed by reduced or absent platelet aggregation to adenosine diphosphate (ADP) (< 50%) and normal to ristocetin (70–100%) upon performing light transmission aggregometry [37]. Blood samples were withdrawn into 2 mL plastic (polypropylene) tubes with 3.2% sodium citrate as an anticoagulant [38]. ADP was used at a concentration of 10 μmol/L, while ristocetin was used at 1.2 mg/L [39, 40] Other conditions causing defective platelet aggregation with ADP, such as acquired platelet function disorders and medication-induced platelet dysfunction, were excluded [41]. Flow cytometry was performed to quantify CD41 and CD61 receptors on the platelet surface via conjugation with fluorescent monoclonal antibodies, classifying GT subtypes. For this study, we defined GT type I as the levels of either CD41 or CD61 being < 5% of normal, GT type II as the levels of either CD41 or CD61 being from 5% to ≤ 50% of normal, and GT type III as the levels of both CD41 and CD61 being > 50% of normal [42]. However, the dysfunction of CD41 and CD61 receptors was not confirmed by DNA sequencing of ITGA2B and ITGB3 genes due to its unavailability in Egypt.

### Disease parameters and clinical manifestations

Bleeding manifestations were reported under 11 categories of bleeds, as recorded in the medical files of PwGT and during the interview surveys with PwGT and their caregivers. Bleeding score was calculated by the treating physician using the International Society on Thrombosis and Haemostasis Bleeding Assessment Tool (ISTH-BAT) [43]. The cut-off values for normal bleeding scores were < 3 in children and adolescents (≤ 17 years), < 4 in adult males, and < 6 in adult females (≥ 18 years) [44].

### Treatment regimens

The available hemostatic therapies were platelet transfusion, recombinant activated factor VII (rFVIIa), and tranexamic acid. In addition, iron and hormonal therapies, as well as previous surgery and its type– when data was available– were reported. Furthermore, the number of hospital admissions and hospitalization days in 2022 were also reported. The treatment and surgical outcomes were not assessed in this study due to data unavailability.

### Ethics committee approval

The study was conducted in accordance with the Declaration of Helsinki. The institutional review board (IRB) of Assiut University approved the study protocol under approval number 04-2023-300263. All participants provided their consent before participation in the study.

### Data summary and statistical analysis

Data collected from each participant was copied into the master data collection spreadsheet using Microsoft Excel software, version 2404. Data summaries were produced for each data category using descriptive statistics. For most categories, the frequency of their reporting was calculated and reported as proportions of the total number of study participants. Quantitative data, such as the patient’s age, the ISTH-BAT score, and the number of hospital admissions per year were calculated and reported as means and standard deviations (SD) using IBM Statistical Package for Social Sciences (SPSS) software version 22. In addition, the relationship between the ISTH-BAT score and CD41 and CD61 levels in children and adolescents (< 18 years), excluding GT type III and the unknown values, was constructed using Pearson’s correlation coefficient (r). The degree of correlation (r) was < 0.20 for no or very weak correlations, from 0.20 to 0.39 for weak correlations, from 0.40 to 0.59 for moderate correlations, from 0.60 to 0.79 for strong correlations, and ≥ 0.80 for very strong correlations [45]. A *p*-value of < 0.05 was considered statistically significant [46].

## Results

### Demographic and disease characteristics of the study group

**Fig. 1:**
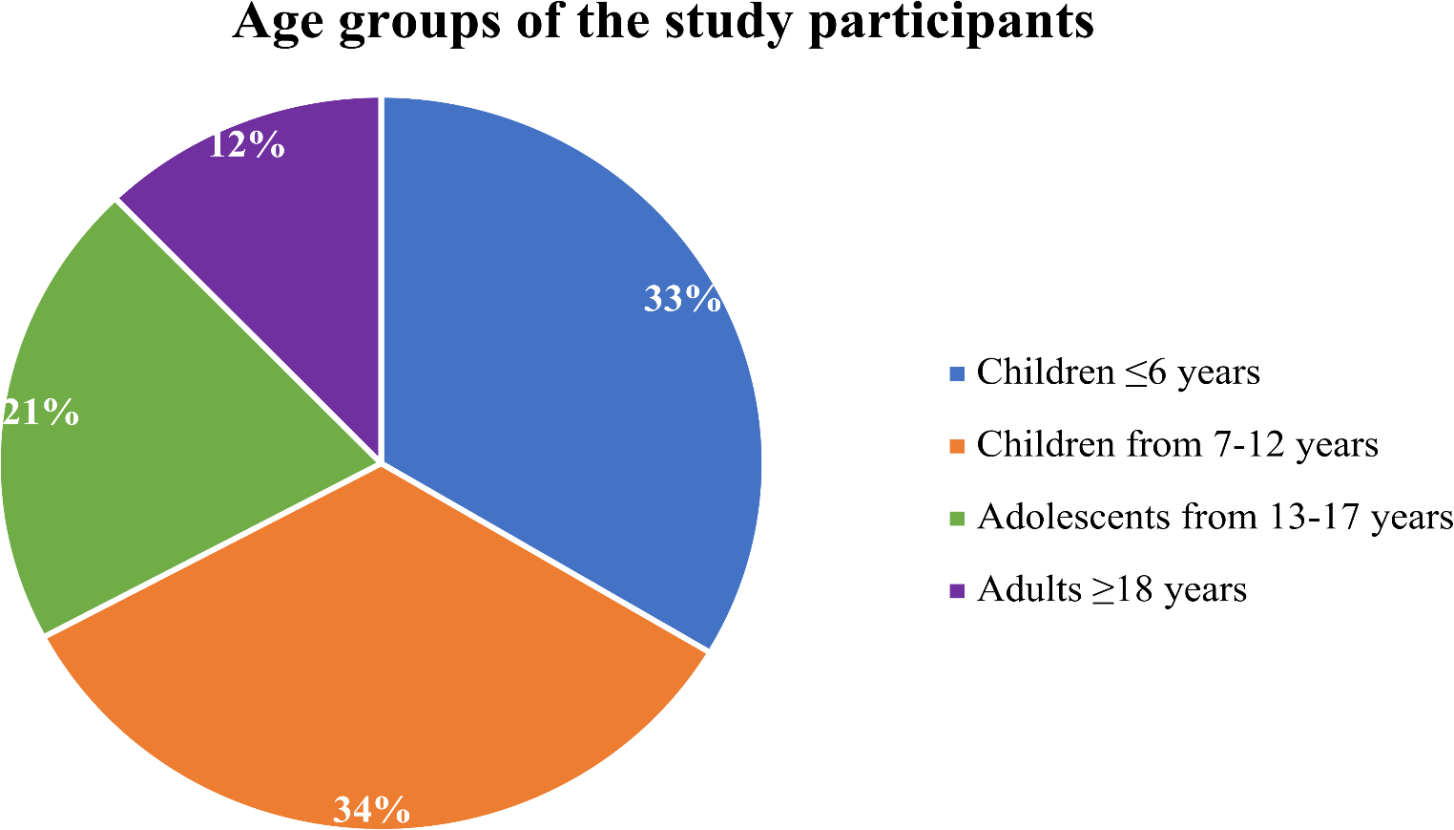
Age groups of the study participants, *n* = 131

We enrolled 131 individuals with GT from different age groups, of whom 88 (67.2%) were children aged 12 years or younger, 27 (20.6%) adolescents aged 13 to 17 years, and 16 (12.2%) were adults aged 18 years or older (Fig. 1). The mean age of the participants was 9.7 ± 5.8 years, with a median age of ten years. Females represented 54.2% of the study participants, while the remaining 45.8% were males. Ninety-six participants (73.3%) of the study cohort had type I GT, 31 (23.7%) had type II GT, and four (3%) had type III GT. Positive consanguinity was present in 114 (87%) of the study participants, while it was absent in the remaining 17 (13%). Family history was positive in 82 (62.6%) of the study cohort and negative in the remaining 49 (37.4%) (Table 1).

**Table 1:** Demographic and disease characteristics of the study participants, *n* = 131

Variable		Assiut *n* = 47	Sohag *n* = 44	Minya *n* = 23	Fayoum *n* = 10	Beni Suef *n* = 7	Total *n* = 131
Age (years), Mean ± SD		9.6 ± 5.6	9.4 ± 6.4	12.6 ± 6.1	8.2 ± 5.4	5.2 ± 4.1	9.7 ± 6.1
Gender	Males, n (%)	19 (40.4%)	21 (47.7%)	10 (43.5%)	6 (60%)	4 (57.1%)	60 (45.8%)
	Females, n (%)	28 (59.6%)	23 (52.3%)	13 (56.5%)	4 (40%)	3 (42.9%)	71 (54.2%)
GT type	Type I, n (%)	29 (61.7%)	44 (100%)	13 (56.5%)	5 (50%)	5 (71.4%)	96 (73.3%)
	Type II, n (%)	18 (38.3%)	0 (0%)	6 (26.1%)	5 (50%)	2 (28.6%)	31 (23.7%)
	Type III, n (%)	0 (0%)	0 (0%)	4 (17.4%)	0 (0%)	0 (0%)	4 (3%)
Consanguinity	Positive, n (%)	43 (91.5%)	35 (79.5%)	23 (100%)	10 (100%)	3 (42.9%)	114 (87%)
	Negative, n (%)	4 (8.5%)	9 (20.5%)	0 (0%)	0 (0%)	4 (57.1%)	17 (13%)
Family history	Positive, n (%)	33 (70.2%)	24 (54.5%)	14 (60.9%)	9 (90%)	2 (28.6%)	82 (62.6%)
	Negative, n (%)	14 (29.8%)	20 (45.5%)	9 (39.1%)	1 (10.0%)	5 (71.4%)	49 (37.4%)

Abbreviations: GT: Glanzmann’s thrombasthenia; SD: Standard deviation

### Laboratory results of the study participants

Platelet aggregation to ADP and ristocetin was performed in all cases. The median value of ADP aggregation was 8%, the mean was 8.7%, and the values ranged from 0 to 30%. In 96 participants with type I GT, CD41 levels ranged from 0 to 23%, while CD61 levels ranged from 0 to 97%. The median values for CD41 and CD61 in type I GT were 0.3% for both, while the mean values were 2% and 5.8%, respectively. In 31 participants with type II GT, CD41 levels had a mean value of 17.4%, the median was 12% and ranged from 5 to 50%, while the mean value of CD61 was 27.5%, the median was 17% and ranged from 5 to 90%. In the remaining four participants with type III GT, all values of CD41 and CD61 were ≥ 90% (Table 2).

**Table 2:** CD41 and CD61 levels stratified by GT type, *n* = 131

	CD41 levels			CD61 levels		
	Type I, *n* = 96	Type II, *n* = 31	Type III, *n* = 4	Type I, *n* = 96	Type II, *n* = 31	Type III, *n* = 4
Mean ± SD	2 ± 4.4%	17.4 ± 13.2%	94 ± 3.5%	5.8 ± 15.1%	27.5 ± 22.2%	96.7 ± 2.6%
Median	0.3%	12%	94.5%	0.3%	17%	97%

Abbreviations: CD: Cluster of differentiation; GT: Glanzmann’s thrombasthenia; SD: Standard deviation

### Clinical characteristics of the study participants

Epistaxis was recorded in 101 out of 131 participants (77.1%), subcutaneous bleeds in 53/131 (40.5%) participants, menorrhagia/heavy menstrual bleeding in 29/131 (22.1%) participants, and mucosal bleeds in 24/131 (18.3%) participants. In a lower occurrence, major organ bleeds were extracted from 9 of 131 medical charts (6.9%), dental bleeds from 3/131 (2.3%), and gastrointestinal bleeds from 2/131 (1.5%) medical charts. In much lower occurrence, bleeds of the central nervous system, hematuria, and ear bleeds were reported in one patient (0.76%) of the study cohort. Nineteen (14.5%) of the study participants underwent 20 surgical procedures, of whom 15 (78.9%) participants were males. The type of surgical procedure was documented for 16/20 (80%) surgeries. All procedures were minor, including adenoidectomy, circumcision, colonoscopy/colon biopsy, inguinal hernia, and lip surgery. The most frequent procedure was circumcision, representing 11/16 (68.8%) of the procedures with a known type.

**Fig. 2:**
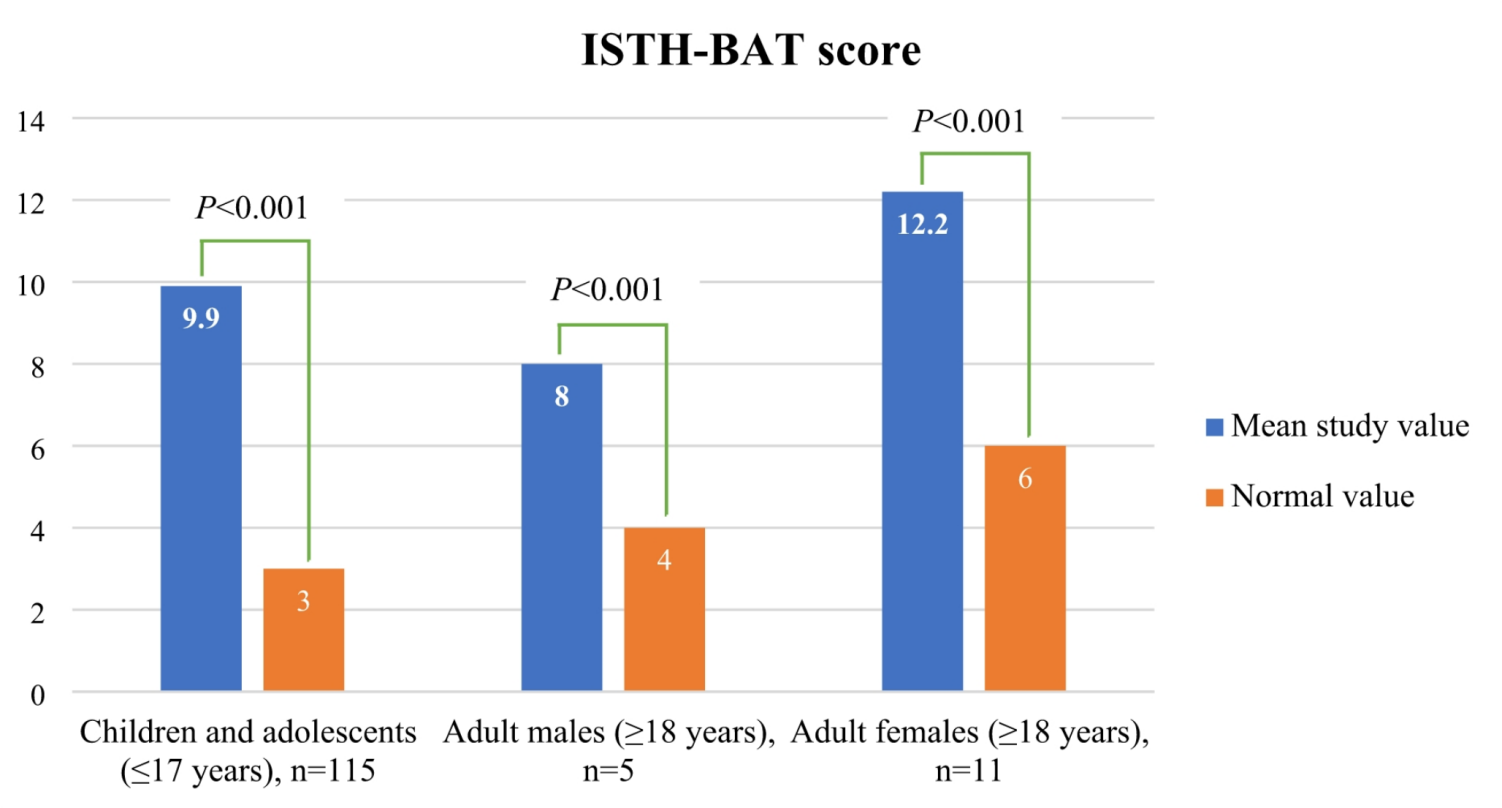
ISTH-BAT score compared to normal in three groups of participants, *n* = 131

**Fig. 3:**
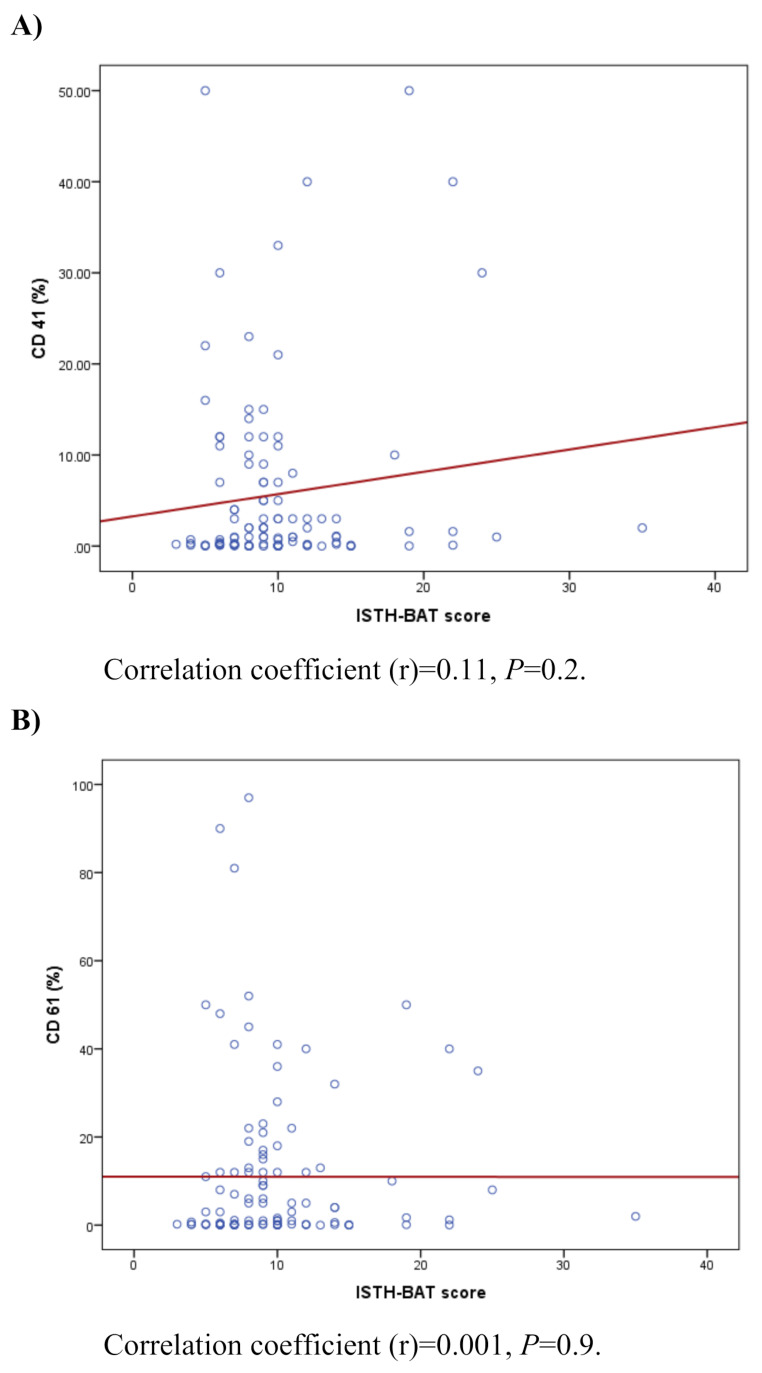
The relationship between the ISTH-BAT score and CD41 (**A**) and CD61 (**B**) levels in children and adolescents (≤ 17 years), *n* = 115

The ISTH-BAT score was abnormal in all participants, with a mean value of 10 ± 4.8 and median of nine. Among the 115 children and adolescents (≤ 17 years), the mean score was 9.9 compared to a normal value of three. In the five adult males (≥ 18 years), the mean score was eight versus four as a normal value. For the 11 adult females, the mean score was 12.2, while the normal value is six. All differences between the observed scores and normal values were highly significant (*p*-value < 0.001) (Fig. 2). The number of hospital admissions in 2022 was available for 129 (98.5%) participants, with a mean value of 3.5, and a median of two admissions per year (Table 3). The range was reported from zero to 16 admissions per year. The number of hospitalization days was recorded in one center (Al-Fayoum) for eight out of 10 participants. The mean and median numbers of hospitalization days, excluding surgeries were 13 days, and ranged from two to 30 days per year. The mean duration of the in-patient stay per hospital admission was 2.2 days, the median was 1.5 days, and the range was from one to four days. The relationship between the ISTH-BAT score and CD41 and CD61 levels was charted employing data from 113 pediatric and adolescent participants (≤ 17 years) with type I and type II GT. Pearson’s correlation coefficient (r) was 0.11 for CD41 and 0.001 for CD61 (Fig. 3).

**Table 3:** Clinical characteristics of the study participants, *n* = 131

Variable	Assiut *n* = 47	Sohag *n* = 44	Minya *n* = 23	Fayoum *n* = 10	Beni Suef *n* = 7	Total *n* = 131
Epistaxis, n (%)	34 72.3%	35 (79.5%)	18 (78.3%)	8 (80%)	6 (85.7%)	101 (77.1%)
SC bleeding, n (%)	25 (53.2%)	17 (38.6%)	4 (17.4%)	6 (60%)	1 (14.3%)	53 (40.5%)
Menorrhagia/ heavy menstrual bleeding, n (%)	15 (31.9%)	9 (20.5%)	4 (17.4%)	0 (0%)	1 (14.3%)	29 (22.1%)
Mucosal bleeding, n (%)	4 (7.8%)	2 (4.3%)	2 (7.4%)	10 (100%)	6 (85.7%)	24 (18.3%)
Major organ bleeding, n (%)	6 (12.8%)	0 (0%)	3 (13%)	0 (0%)	0 (0%)	9 (6.9%)
Dental bleeding, n (%)	0 (0%)	0 (0%)	0 (0%)	3 (30%)	0 (0%)	3 (2.3%)
GI bleeding, n (%)	0 (0%)	0 (0%)	0 (0%)	2 (20%)	0 (0%)	2 (1.5%)
CNS bleeding, n (%)	0 (0%)	0 (0%)	0 (0%)	1 (10%)	0 (0%)	1 (0.76%)
Hematuria, n (%)	0 (0%)	0 (0%)	0 (0%)	1 (10%)	0 (0%)	1 (0.76%)
Ear bleeding, n (%)	0 (0%)	0 (0%)	0 (0%)	1 (10%)	0 (0%)	1 (0.76%)
Previous surgery performed, n (%)	5 (10.6%)	1 (2.3%)	6 (26.1%)	5 (50%)	2 (28.6%)	19 (14.5%)
ISTH-BAT score, mean ± SD	9.1 ± 2.2	8.5 ± 3.7	8.8 ± 1.6	15.6 ± 5.7	21.6 ± 7.4	10 ± 4.7
No. of hospital admissions/year, mean ± SD	5.2 ± 3.2	0.84 ± 1.1	4.7 ± 4.3	6.1 ± 4.3	2.7 ± 1.8	3.5 ± 3.5

Abbreviations: CNS: Central nervous system; GI: Gastrointestinal; ISTH-BAT: the International Society on Thrombosis and Haemostasis Bleeding Assessment Tool; No.: Number; SC: Subcutaneous; SD: Standard deviation

### Type of therapies of the study participants

Ninety-five (72.5%) and 91 (69.5%) out of 131 participants used rFVIIa and platelet transfusion, respectively for episodic treatment of bleeding events and to prevent bleeding during surgical interventions. Both treatment options were administered in the hospital, as home treatment was not feasible due to economic and logistical challenges. While 118/131 (901%) participants used tranexamic acid as an adjunctive therapy to rFVIIa or platelets, 9/131 (6.9%) participants used it as a monotherapy, and 4/131 (3%) participants did not use it at all. Iron therapy was taken by 101/131 (77.1%) participants to treat iron deficiency anemia as a complication of the frequent bleeds in PwGT. In addition, hormonal therapy was prescribed to 12/131 (9.2%) participants (Table 4).

**Table 4:** Type of therapies of the study participants, *n* = 131

Variable	Assiut *n* = 47	Sohag *n* = 44	Minia *n* = 23	Fayoum *n* = 10	Beni Suef *n* = 7	Total *n* = 131
rFVIIa	31 (66%)	40 (90.9%)	9 (39.1%)	8 (80%)	7 (100%)	95 (72.5%)
Platelet transfusion	25 (53.2%)	40 (90.9%)	10 (43.5%)	9 (90%)	7 (100%)	91 (69.5%)
Tranexamic acid as an adjunctive therapy	42 (89.4%)	44 (100%)	16 (69.6%)	9 (90%)	7 (100%)	118 (90.1%)
Tranexamic acid as a monotherapy	4 (8.5%)	0 (0%)	4 (17.4%)	1 (10%)	0 (0%)	9 (6.9%)
Iron therapy	35 (74.5%)	35 (79.5%)	15 (65.2%)	9 (90%)	7 (100%)	101 (77.1%)
Hormonal therapy	5 (10.6%)	4 (9.1%)	3 (13%)	0 (0%)	0 (0%)	12 (9.2%)

Abbreviations: rFVIIa: Recombinant activated factor VII

## Discussion

In this study, we report a summary of epidemiological, sociodemographic, diagnostic, and clinical information collected retrospectively from 131 individuals diagnosed with congenital GT who were following up at five tertiary hospitals in Upper Egypt (Fayoum, Beni-Suef, Minia, Assiut, and Sohag University hospitals) from October 2023 to April 2024. We also reported the type of bleeding manifestations and the current treatment modalities in the studied cohort. Diagnosis was made independently at each center, based on laboratory tests conducted at various local laboratories, the patient’s area of residence and the availability of the requested analyses. A single-center study in Assiut University that included 40 children (1–10 years) with GT was previously published [35].

Our cohort consisted of children and young adults because all the sites included were pediatric hematology centers. The mean age of study participants was 9.7 ± 5.8 years and ranged from six months to 25 years. 67% of the study group were children aged 12 years or younger, while the remaining 33% were adolescents and adults aged 13 years or older. Bleeding manifestations were discovered at an early age, especially after traumatic injuries and during surgical interventions [47]. Data from three studies revealed that approximately, 53% of PwGT experience the first bleed by the age of one year [14, 24, 47], with a median age of diagnosis below two years and nine months [23, 26, 27, 48] (Table 5).

**Table 5:** Comparison of demographic and clinical characteristics of our cohort with other studies

Parameter	This study	George et al., 1990	Al-Barghouthi et al., 1997	Toogeh et al., 2004	Borhany et al., 2012	Kutlubay et al., 2012	Farsinejad et al., 2013	GTR (1,2)	Iqbal et al., 2016	Elmahmoudi et al., 2017	Mutreja et al., 2017	Zhou et al., 2018	Kongalappa et al., 2019	Duncan et al., 2020	Mahmood et al., 2022	Usman et al., 2024
Country	Egypt	France	Saudi Arabia	Iran	Pakistan	Turkey	Iran	15 countries	Pakistan	Tunisia	India	China	India	USA	Pakistan	Pakistan
No. of particip.	131	177	16	382	43	19	95	216	163	27	51	97	48	45	327	103
Age of particip.	9.8 yr*	All AGs	≤l4 yr	≤ 45 yr	≤ 30 yr	≤ 9.83 yr^#^	1–45 yr	All AGs	7 yr*	22 yr^#^	4 yr^#^	1–53 yr	≤ 15 yr	≤ 61 yr	9.17 yr^#^	1.1 yr^#^
% of females	54.2%	57.6%	44%	46.6%	53.5%	42%	54.7%	57.4%	43.6%	51.9%	47%	54.6%	41.7%	58%	65.75%	46.6%
% of + ve consang.	87%	39%^¶^	93%	86.6%	NR	73.7%	93.7%	NR	65%	63%	27.5%	18.3%	66.7%	NR	85.3%	83.5%
% of + ve FH	62.6%	NR	75%	NR	60%	26.3%	NR	NR	83%	48.1%	11.8%	NR	20.8%	31%	28.1%	57.2%
% of type I	73.3%	78%^¶^	NR	NR	NR	36.8%	77.9%	28.3%^§^	NR	NR	47%	74.2%	NR	NR	NR	NR
% of type II	23.7%	14%	NR	NR	NR	31.6%	13.7%	10.9%^§^	NR	NR	11.8%	23.7%	NR	NR	NR	NR
% of type III	3%	8%	NR	NR	NR	10.5%	8.4%	4.3%^§^	NR	NR	41.2%	2.1%	NR	NR	NR	NR
% of unknown type	0%	0%	NR	NR	NR	21%	0%	56.5%^§^	NR	NR	0%	0%	NR	NR	NR	NR
Age at diagnosis	NR	NR	NR	8 yr*	NR	10 mon.^#^	2.86 yr*	NR	NR	1.5 yr^#^	NR	NR	2.75 yr^#^	2.6 yr*	9.17 yr^#^	1.1 yr^#^
Age at the time of 1st BL	NR	NR	NR	NR	NR	9 mon.^#^	NR	1 yr^#§^	NR	NR	NR	NR	1.5 yr^#^	NR	4.25 yr^#^	NR
% of particip. w. epistaxis	77.1%	72.9%	88%	49.7%	67.4%	36.8%	31%	79.2%	62.5%	77%	59%	69.1%	77%	NR	78.6%	71%
% of particip. w. SC BL	40.5%	85.9%	75%	15.1%	58.1%	31.6%	31.6%	43.1%	76.6%	55%	53%	69.1%	62.5%	NR	85%	61%
% of particip. w. muco. BL	18.3%	54.6%	62.5%	22.8%	44.1%	10.5%	8.8%	61.9%	56.4%	81%	49%	36.1%	50%	NR	56.3%	55%
% of pub. females w. menor.	88.9%	98.2%	57.1%	12.9%	100%	NR	57%	73.6%	70%	50%	89%	90.6%	100%	NR	NR	60%
ISTH-BAT score	10*	NR	NR	NR	NR	NR	WHO score	NR	NR	NR	WHO score	WHO score	NR	9.07*	NR	NR

*mean, ^#^median, ^¶^calculated for 64 patients, ^§^calculated for 184 patients, +ve positive, % proportion, AGs age groups, BL bleed(ing), consang. consanguinity, FH family history, ISTH-BAT International Society on Thrombosis and Haemostasis Bleeding Assessment Tool, menor. menorrhagia, mon. month(s), muco. mucosal, No. number, NR not reported, particip. participants, pub. pubertal, SC subcutaneous, USA United States of America, WHO World Health Organization, w. with, yr year(s)

The gender distribution in our cohort was slightly inclined towards female participants, representing 54.2% of the total compared to male participants at 45.8%. This little skewing was mainly driven by the greater proportion of females (62.8%) compared to males (37.2%) in the adolescent and adult age groups in our cohort although females represent only 48.6% of the Egyptian general population [49]. Among those 27 females, 24 (88.9%) experienced menorrhagia, which was their most common hemostatic challenge, followed by epistaxis and subcutaneous bleeding, which were experienced by 13 (48.1%) and four (14.8%), respectively. This is in line with the prevalence of menorrhagia among pubertal females in the reproductive age group reported in previously published reports, ranging from 70 to 100% [13, 14, 19, 22, 25, 26, 34, 47, 50], except for a study conducted in Tehran, Iran that reported a much lower proportion at just 12.9% [33]. However, in a more recent study conducted in the same city, 57% of reproductive women reported menorrhagia [24]. The discrepancy in menorrhagia rates across different studies may highlight variability in the location and severity of bleeding manifestations in GT [42, 47]. It may also be influenced by sociocultural barriers that hinder the actual reporting of menorrhagia in various populations [51–53]. Among the 29 young females, including five girls aged from 11 to 12 years, who reported having heavy menstrual bleeding in our cohort, 12 (41.4%) were using hormonal therapies, indicating they experienced heavy menstruation that interrupted their daily activities and impacted their overall quality of life [32, 54].

Approximately 97% of this study participants were diagnosed with type I and type II GT. This proportion was mostly matched with that of a study conducted in East China, in which 97.9% of the study population diagnosed with type I and type II GT [34]. In a previous study conducted in Assiut, 80% of participants were reported to have type I and type II GT. In contrast, all participants recruited from Assiut in this study were diagnosed with either type I and type II GT. This difference was mainly attributed to the lower proportion of patients with type I GT (the most severe form) in the earlier study [35].

Alternatively, we might have overestimated the proportion of people with type III GT, representing 3% of our cohort because of the potential overlap in diagnosis with type II GT [42] and the limited laboratory and molecular diagnostic tools at our centers [2, 3]. If these tools were accessible, we could have identified more individuals with type III GT and the proportion could have reached 10%, as estimated by Poon et al. [47]. Clot retraction, a simple clotting technique might have helped us differentiate between some inconclusive cases with type II and type III GT in the absence of molecular analysis in our centers [42, 55]. We excluded 12 participants with an unknown GT type from our study, as their flow cytometry results were unavailable. This was done to avoid the potential misdiagnosis that might occur with other inherited platelet disorders caused by signal transduction defects, such as kindlin 3 or CalDAG-GEFI deficiencies, which exhibit similar platelet aggregation results with ADP and ristocetin [40, 56–58]. Interestingly, the proportion of participants with an unknown GT type exceeded 56% in an international GT Registry (GTR) that included 216 patients with GT [11, 47].

Table 1 shows significant variations in the proportions of GT types reported from the included centers. For example, all participants in Sohag were diagnosed with type I GT, while Fayoum and Minya reported these proportions at 50% and 56.5%, respectively. In addition, all centers except Al-Minya recruited people with type III GT. Without performing molecular testing, we could not be sure whether these differences relate to specific gene mutations in each governorate or were due to inaccurate diagnoses.

Platelet aggregation to ADP was absent up to low normal (≤ 30%) in all participants (**Data not shown**). We excluded suspected individuals with GT whose platelet aggregation to ristocetin was < 70%. The mean values of CD41 and CD61 levels in our cohort were 2% and 5.8% in type I GT, 17.4% and 27.5% in type II, as well as 94% and 96.7% in type III. Slightly higher values for CD41 in type II only and CD61 in both type I and type II were observed in a GT cohort in New Delhi, India [13]. In our study, six patients had CD61 values above 50%, while their disease type was classified as type I or II. This classification relied on the condition in our methodology, in which both CD41 and CD61 values should score ≤ 50% to be designated as type I or II GT [42]. Nevertheless, similar cases were reported in previous studies, such as cases number three and 15 in a study conducted in Pakistan [29].

87% of PwGT enrolled in our cohort were the offspring of first- and second-cousin consanguineous marriages, while no discernible inheritance pattern was found in 13% of our group. Likewise, high consanguinity rates were also observed in Iran, Pakistan, Saudi Arabia, South India, Tunisia, and Turkey, ranging from 63 to 98% [14, 20, 22–24, 26–29, 31, 33]. On the contrast, lower consanguinity rates were reported from East China, North India, and Paris, France, ranging from 18.3 to 39% [13, 25, 34]. The high rates of consanguinity in certain regions and among specific ethnic groups may increase the prevalence of GT to reach that of more prevalent bleeding disorders, such as hemophilia and von Willebrand disease [25, 31]. In addition, the long-standing consanguinity in Upper Egypt [59] makes it difficult to know whether the disease is caused by inherited or de novo mutations unless a molecular diagnosis is implemented [60, 61]. Nonetheless, up to 500 genetic mutations in the ITGA2B and ITGB3 genes have been reported in various geographical locations and among ethnic groups [29, 61–67].

Almost 63% of the enrolled PwGT reported a positive family history of GT in our study. Comparable and higher proportions of PwGT with known family history of GT were reported from Pakistan and Saudi Arabia, ranging from 57.2 to 86% [14, 20, 22, 31, 48]. Other studies conducted in India, Pakistan, Turkey, Tunis, and USA reported lower proportions of a positive family history, ranging from 11.8 to 48.1% [13, 23, 26–28, 68].

Epistaxis was the most frequently reported bleeding manifestation in our cohort, observed in 77% of participants. This figure is comparable to that found in four previous studies, ranging from 77 to 79.2% [23, 26, 28, 47]. A higher proportion of 88% was reported by a single study conducted in East Saudi Arabia [20]. In contrast, 77% of GT patients in two cohorts from Tunisia and Southern India manifested with epistaxis nine previous studies reported lower proportions of epistaxis, ranging from 31 to 73% [13, 14, 22, 24, 25, 27, 33, 34, 48]. The second most reported bleeding manifestation in our cohort was subcutaneous bleeding, which was experienced by 40.5% of the study population. Eleven previous studies reported higher proportions of subcutaneous bleeding, ranging from 53 to 85.9% [13, 14, 20, 22, 23, 25, 26, 28, 34, 47, 48]. On the other hand, three other studies reported lower values, ranging from 15.1 to 31.6% [24, 27, 33].

Mucosal bleeds, including oral cavity and gingival bleeds, were less common in our cohort and occurred in 18.3% of participants. Higher proportions of gum bleeds, ranging from 22.8 to 81% of participants were reported in previously published studies from various parts of the world [13, 14, 20, 22, 23, 25, 26, 28, 33, 34, 47, 48]. On the other hand, lower figures were reported from two previous studies conducted in Iran and Turkey [24, 27] (Table 5).

Major organ bleeds were reported by 6.9%, dental bleeds by 2.3%, and gastrointestinal bleeds by 1.5% of the study participants. Central nervous system and ear bleeds, as well as hematuria, were recorded in < 1% of the patient’s medical files. Twenty minor surgical interventions were performed in 14.5% of PwGT recruited in our study. Among the 16 surgeries for which the type was known, circumcision was the most frequent procedure (68.8%), performed in 11 participants.

All participants had abnormal ISTH-BAT scores, which showed highly significant differences from normal values based on age group and gender, as illustrated in Fig. 2. The relationship between the ISTH-BAT score and CD41 and CD61 levels in children and adolescents with type I and type II GT was found to be very weak and non-existent, respectively, indicating that the residual levels of the deficient integrin αIIbβ3 did not correlate with the bleeding severity and frequency in our cohort (Fig. 3). Such a discrepancy between the clinical phenotype and the biological activity of the deficient integrin αIIbβ3 in PwGT was previously reported [42, 47], and any bleed in PwGT can be severe or life-threatening, which occurs unexpectedly over their lifespan [69]. Those with type III GT were excluded from this analysis because their CD41 and CD61 levels do not correlate with their clinical severity. In addition, we excluded adult participants (≥ 18 years) from this analysis because they have different normal values compared to children and adolescents (≤ 17 years), as well as based on their gender [44].

The mean number of hospital admissions across all centers was 3.5 per year. In a single center, the mean length of hospital stay, excluding perioperative periods, was 13 days per year and 2.2 days per hospital admission. To our knowledge, this is the first study reporting such inpatient-related information. Nonetheless, the findings would be more robust if information on the length of hospitalization was collected from all participating centers.

More than 93% of participants in this study used rFVIIa and/or platelet transfusion for episodic treatment of bleeding events and surgical coverage. Tranexamic acid was used as an adjunctive therapy by more than 90% of the total cohort, while approximately 7% used it as a monotherapy, which could be explained by the moderate to severe bleeding pattern of PwGT in our cohort [70]. Only 3% of participants did not use tranexamic acid at all.

More than 77% of participants received iron replacement therapy, administered as intravenous injections during hospital admissions and as oral therapy for the outpatient use. This treatment was mainly given due to chronic anemia, resulting from recurrent blood loss or insufficient iron intake to meet their higher needs. Additionally, dietary factors and other variables can worsen iron deficiency, especially in the presence of this chronic bleeding disorder [71].

Currently, there is no comprehensive national registry for GT in Egypt, making it challenging to estimate the actual burden of the disease in Upper Egypt compared to the rest of the country. However, Egypt comes second worldwide in the number of people diagnosed with GT, with 502 cases, as reported in the World Federation of Hemophilia Report on the Annual Global Survey 2022 [72]. In addition, GT is the fourth most prevalent inherited bleeding disorder in Egypt, following hemophilia A and B and von Willebrand disease [72]. Based on a population of 105,914,499 in 2024 [49], the prevalence proportion of GT in Egypt roughly equals one per 200,000 or five per million people [73]. Although the precise incidence and prevalence of GT have proven challenging to determine, the global estimates are approximately one per million people [2, 4]. However, these proportions increase in communities with high consanguinity rates [3] and may exceed the global average, such as in some Arabian Gulf countries [32]. High rates of consanguinity are also observed in Egypt, especially in rural governorates, including Upper Egypt, where the proportion of consanguineous marriage may surpass 42% in some governorates [59, 74, 75]. Therefore, GT prevalence is expected to be greater than one per 200,000 in rural areas in Egypt, as previously reported [23]. Based on a population of 25,034,120 in the five governorates included in our study [49], the prevalence of GT likely exceeds this figure and may increase with improved diagnosis and the identification of additional cases [73].

### Strengths and limitations

Our study has some strengths, such as being the first report from Egypt on GT, involving multiple centers. In addition, the number of PwGT enrolled in our study is relatively large, which is significant given the rarity of GT. Furthermore, we compared our results to previously published studies from various parts of the world. On the contrary, it has some limitations, such as the retrospective nature of the study design, which relies on non-systematic recording of medical data in patients’ files [76, 77]. As a result, we could not capture some important information, such as the age at diagnosis and the age at the first bleeding episode, as well as the frequency of bleeding episodes in PwGT within a specific timeframe. This information would be valuable for comparing the clinical characteristics of our cohort with those from other studies. In addition, we have not assessed or compared the treatment outcomes for different treatment options or during surgery in our study. Furthermore, some laboratory tests were not comprehensively performed in our cohort, such as molecular testing to confirm the clinical diagnosis. Moreover, drawing a relationship between the residual levels of CD41 and CD61 and the ISTH-BAT score in people with type I and type II GT was affected by the accuracy of measuring CD41 and CD61 levels by flow cytometry.

## Conclusion

Rare hereditary bleeding disorders, such as GT require the collection of comprehensive and accurate medical information to support estimating the actual disease burden. The prevalence of GT seems to be higher in Upper Egypt than the national average due to complex consanguinity among families in various regions. We encourage primary care physicians to refer cases with suspected bleeding manifestations to hematology-specialized centers to identify potential cases with GT. However, social and cultural barriers often lead many individuals, particularly women with heavy menstrual bleeding, to ignore their symptoms, resulting in significant underreporting of menorrhagia among females. The diagnostic workup for GT includes platelet aggregation, clot retraction, and flow cytometry, which requires the involvement of laboratory specialists in making the diagnosis of PwGT. However, the accuracy of disease subtyping is hampered by the high cost of molecular analysis, which is often prohibitively expensive in Egypt. In our study, we mapped the epidemiological, sociodemographic, diagnostic, and clinical characteristics of a large cohort of PwGT. We hope this information will serve as a foundation for a national registry for all PwGT in Egypt in the future.

## Data Availability

No datasets were generated or analysed during the current study.
